# Vasodilation of Tea Polyphenols Ex Vivo Is Mediated by Hydrogen Peroxide under Rapid Compound Decay

**DOI:** 10.3390/antiox9050390

**Published:** 2020-05-07

**Authors:** Mario Lorenz, Stephanie Lehmann, Ilija Djordjevic, Thomas Düsterhöft, Benno F. Zimmermann, Karl Stangl, Verena Stangl

**Affiliations:** 1 Charité – Universitätsmedizin Berlin, Corporate Member of Freie Universität Berlin, Humboldt-Universität zu Berlin, and Berlin Institute of Health, Medizinische Klinik für Kardiologie und Angiologie, Campus Mitte, 10117 Berlin, Germany; thomdue@yahoo.de (T.D.); karl.stangl@charite.de (K.S.); verena.stangl@charite.de (V.S.); 2 DZHK (German Centre for Cardiovascular Research), Partner Site Berlin, 10115 Berlin, Germany; 3 Fachbereich Veterinärmedizin, Institut für Veterinär-Biochemie, Freie Universität Berlin, 14163 Berlin, Germany; s.lehmann1911@web.de; 4 Klinik und Poliklinik für Herzchirurgie, Herzchirurgische Intensivmedizin und Thoraxchirurgie, Universitätsklinikum Köln, 50937 Köln, Germany; ilija.djordjevic@uk-koeln.de; 5 Institut für Ernährungs- und Lebensmittelwissenschaften, Universität Bonn, 53115 Bonn, Germany; benno.zimmermann@uni-bonn.de

**Keywords:** EGCG, theaflavin, polyphenols, green tea, black tea, vasodilation, ROS, hydrogen peroxide

## Abstract

Improvement of endothelial function represents a major health effect of tea in humans. Ex vivo, tea and tea polyphenols stimulate nitric oxide (NO)-dependent vasodilation in isolated blood vessels. However, it was reported that polyphenols can generate reactive oxygen species (ROS) in vitro. We therefore aimed to elucidate the role of ROS production in tea polyphenol-induced vasodilation in explanted aortic rings. Vasorelaxation of rat aortic rings was assessed in an organ chamber model with low concentrations of epigallocatechin-3-gallate (EGCG), theaflavin-3,3’-digallate (TF3), and with green and black tea, with or without pretreatment with catalase or superoxide dismutase (SOD). The stability of EGCG and TF3 was measured by HPLC, and the levels of hydrogen peroxide (H_2_O_2_) were determined. EGCG and green tea-induced vasorelaxation was completely prevented by catalase and slightly increased by SOD. TF3 and black tea yielded similar results. Both EGCG and TF3 were rapidly degraded. This was associated with increasing H_2_O_2_ levels over time. Hydrogen peroxide concentrations produced in a time range compatible with tea polyphenol decay induced NO-dependent vasodilation in aortic rings. In conclusion, tea polyphenol-induced vasodilation in vitro is mediated by low levels of H_2_O_2_ generated during compound decay. The results could explain the apparent lack of vasodilatory effects of isolated tea polyphenols in humans.

## 1. Introduction

Plant-derived polyphenols are widely considered as beneficial for human health [1]. Tea contains high amounts of polyphenols and consumption of tea has been attributed to many health-promoting effects [2,3,4]. Diseases of the cardiovascular system are particularly alleviated by tea intake [5,6]. High consumption of tea is associated with reduced cardiovascular mortality [7,8,9] and with lower progression of atherosclerosis [10]. Catechins, especially epigallocatechin-3-gallate (EGCG), are presumed to mediate the health-promoting effects of green tea [11]. A reduction in atherosclerosis progression has been shown for green tea and EGCG in animal experiments [12,13]. Impairment of endothelial function, measured as disturbed flow-mediated dilation (FMD), is an established early marker of atherosclerosis [14,15]. Improvement of FMD represents a well-known cardiovascular feature of tea polyphenols. Many studies have shown an increase in FMD after consumption of tea [16], and green and black tea resulted in comparable effects [17]. In black tea, the catechins are converted to higher molecular theaflavins and thearubigins during fermentation [18].

Elucidation of the underlying mechanisms for tea polyphenol-induced vasodilation and identification of individual tea compounds have made substantial progress in recent years. In isolated aortic rings, green and black tea stimulated nitric oxide-dependent vasodilation [19]. EGCG and theaflavin-3,3′-digallate (TF3) (among other black tea polyphenols) induced a concentration-dependent vasorelaxation that was prevented by denudation of the endothelium or by inhibition of nitric oxide (NO) production [19,20]. The above results indicate an endothelial- and NO-dependent mechanism for the vasodilatory effects of tea polyphenols. Surprisingly, we observed that EGCG is not involved in tea-induced improvement of flow-mediated dilation in humans [21]. In the present study, we therefore aimed to elucidate the apparent discrepancy for tea polyphenol-mediated vasodilation between humans and explanted organs in vitro. Green tea catechins were stable in acidic solutions, but unstable at higher pH values [22,23]. Similar findings were obtained for green tea catechins as part of a green tea extract [24]. Polyphenols from black tea were also unstable at higher pH values [25]. It was reported that tea polyphenols can produce reactive oxygen species (ROS) under certain cell culture conditions [26]. To study the mechanisms of tea polyphenol-induced vasodilation in vitro, we performed experiments with and without antioxidant enzymes in aortic rings. We measured compound stability and hydrogen peroxide production. To get a broader insight, both green and black tea polyphenols were included.

## 2. Materials and Methods

### 2.1. Animals

Male Wistar rats (300–350 g) from Charles Rivers Laboratories (Germany) were used for the experiments. The animals were kept according to institutional guidelines under a standard diet and water ad libitum. Extraction of organs from the animals was approved by the local authority (Landesamt für Gesundheit und Soziales, Berlin) under the permit number T0026/05.

### 2.2. Preparation of Tea and Tea Polyphenols

EGCG was obtained from Sigma (Deisenhofen, Germany) and TF3 was kindly provided by Mitsui Norin Food Research Laboratories (Fujieda-shi, Japan). Green and black Assam tea was provided by King’s Teagarden (Berlin, Germany). Tea were brewed in 500 mL of boiling water for 3 min using 6 g (green tea) or 5 g (black tea) of tea leaves. The concentration of EGCG in green tea was 1031 µM and of TF3 in black tea was 11.1 µM, which was determined prior to the experiments by HPLC.

### 2.3. Experimental Procedure of Vasorelaxation Studies with Aortic Rings

Thoracic aortas from healthy male Wistar rats were rapidly excised, cleaned of the surrounding tissue and cut into rings of 2 mm length under sterile conditions. The measurement of changes in the vasoreactivity of the explanted aortic rings were performed in an organ chamber. The rings were mounted on platinum hooks in 10 mL jacketed organ baths containing a modified Krebs–Henseleit solution (144 mM NaCl, 5.9 mM KCl, 1.6 mM CaCl_2_, 1.2 mM MgSO_4_, 1.2 mM KH_2_PO_4_, 25 mM NaHCO_3_, 11.1 mM D-glucose) and 1 µM diclofenac. The solution in the bath was maintained at a pH of 7.4 and at 37 °C, with a gas mixture of 5% CO_2_ and 95% O_2_. After equilibration, the reactivity of rings was tested with KCl (40 mM). Non-functional rings were discarded. Rings were precontracted with phenylephrine (PE, 0.05 µM) before treatments.

### 2.4. Treatments of Aortic Rings

Relaxation was stimulated with cumulative doses of EGCG (0.1–10 µM) or TF3 (0.02–2 µM). Green and black tea were applied to the aortic rings at 5–50 µL concentrations. All vasorelaxation treatments were carried out at 30 min intervals. Rings with the same amount of water served as controls. Selected rings were preincubated with 200 U/mL catalase or with 500 U/mL superoxide dismutase (both from Sigma) before precontraction with phenylephrine. Experiments with hydrogen peroxide (H_2_O_2_) were performed with or without the NOS inhibitor L-NAME (N-nitro-L-arginine methyl ester, 0.1 mM), applied before phenylephrine exposure. Vasorelaxation was expressed as the percentage of precontraction with phenylephrine. The data represent numbers of individual aortic rings. All experiments were done with at least 3 different animals.

### 2.5. Determination of Tea Polyphenol Concentrations by HPLC

To determine concentrations of individual tea polyphenols in the beverages, green and black tea were diluted with 10% acetonitrile in water containing 500 µg/mL EDTA and ascorbic acid. Concentrations of EGCG and TF3 were measured on a Waters Acquity UPLC (Waters, Milford, MA, USA). The equipment consists of a binary pump (BSM), an autosampler (SM) cooled at 10 °C, a column oven (CM) set at 40 °C, a diode array detector (PDA) scanning from 190 to 500 nm, and an Acquity TQD triple-quadrupole mass spectrometer with an electrospray interface. A Waters BEH phenyl column (50 mm x 2.1 mm, 1.7 µm) with a VanGuard precolumn was employed at a flow rate of 0.6 mL/min. The eluents acetonitrile/0.1% formic acid (A) and water/0.1% formic acid (B) were run with the following gradient: 0 min: 6% A; 1.5 min: 13% A; 3.0 min: 20.5% A; 4.5 min: 42.5% A; 4.8–5.5 min: 100% A; 5.8–6.3 min: 6% A. EGCG and TF3 were quantified by external calibration with pure EGCG (Sigma-Aldrich, Steinheim, Germany) and theaflavin-3,3’-digallate (LGC Standards, Wesel, Germany) as references using UV detection at 278 nm. Peak identity was confirmed by MS/MS. Liquid samples were adequately diluted with methanol/water (80/20), and filtered through 0.2 µm Chromafil RC-20/15 MS filters (Macherey-Nagel, Düren, Germany).

For the measurement of the stability of tea polyphenols, single doses of EGCG (1 and 10 μM) and TF3 (0.5 and 2 μM) were applied to the Krebs–Henseleit solution and gassed as above. Aliquots (1 mL) were collected after different time points (1, 15 and 30 min). Samples were immediately adjusted to a pH of 3.4 with hydrochloric acid and frozen at −80 °C to stabilize the tea polyphenols. Experiments were performed with or without 200 U/mL catalase or 500 U/mL superoxide dismutase. Compound concentrations were determined as above.

### 2.6. Measurement of Hydrogen Peroxide Levels

Single doses of EGCG (1 and 10 μM), TF3 (0.5 and 2 μM) and of green and black tea (50 µL each) with or without 200 U/mL catalase or 500 U/mL superoxide dismutase were applied. Aliquots of the Krebs–Henseleit solution (200 µL) were collected after 1, 15 and 30 min. Krebs–Henseleit buffers without treatment served as the controls. The samples were treated with 200 μM acetanilide to prevent H_2_O_2_ decay. Concentrations of hydrogen peroxide were measured with the Amplex Red Hydrogen Peroxide Assay (Invitrogen) according to the instructions of the manufacturer. Horseradish peroxidase (HRP) catalyzes the conversion of the Amplex Red Reagent (NAcetyl-3,7-dihydroxyphenoxazines; colorless, non-fluorescent) in a stoichiometric 1:1 reaction with H_2_O_2_ to the red-fluorescent oxidation product resorufin. The optical density of the reaction product was measured at 530 nm using a microplate reader (Molecular Devices, USA). H_2_O_2_ levels were quantified against a standard curve. The detection limit of the kit is 50 nM H_2_O_2_.

### 2.7. Statistical Analysis

Values are given as means ± SEM. Statistical calculations were performed by one-way ANOVA. After the overall statistical differences between the treatments were calculated, post-hoc Tukey-tests were used to adjust for multiple testing. Significance was accepted at a *p*-value < 0.05. Statistical analysis was performed using SPSS, release 22.0 (SPSS, Inc., Chicago, IL, USA).

## 3. Results

### 3.1. Tea Polyphenol-Induced Vasodilation is Prevented by Catalase but Not by SOD

**Figure 1:**
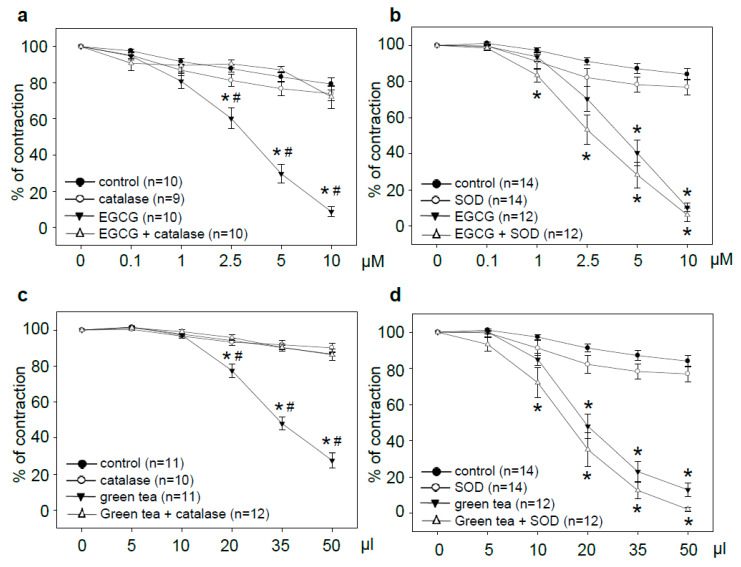
Green tea polyphenol-induced vasorelaxation was inhibited by catalase but not SOD. Vasorelaxation with cumulative doses of EGCG (**a,b**) or green tea (**c,d**) with or without 200 U/mL catalase or 500 U/mL superoxide dismutase (SOD). Graphs show relaxation expressed as the percentage of maximal phenylephrine-induced vasoconstriction. Control rings received the same amount of water (control) or antioxidant enzyme alone. Data are means ± SEM of the indicated number of experiments. * *p* < 0.05 compared to the control; # *p* < 0.05 compared to treatment + catalase.

EGCG produced dose-dependent vasorelaxation in rat aortic rings during a period of 2.5 h. Vasodilation reached significance at 2.5 µM, and almost complete relaxation was achieved at 10 µM. Pretreatment of rings with catalase completely blocked EGCG-induced vasorelaxation. Catalase alone had no impact (Figure 1a). Pretreatment of aortic rings with superoxide dismutase (SOD) slightly potentiated EGCG-induced vasorelaxation. Addition of SOD to control rings resulted in a small reduction of PE-induced contraction (Figure 1b). To determine whether the effects of the antioxidant enzymes are limited to individual compounds, experiments with green tea were performed. Treatment with green tea resulted in concentration-dependent vasorelaxation, reaching significance at 20 µL. Application of 50 µL green tea to the aortic rings resulted in a final concentration of 5 µM EGCG. Catalase completely prevented green tea-induced vasorelaxation (Figure 1c). Pretreatment with SOD caused a small increase in green tea-induced vasodilation that was also observed in the control rings (Figure 1d).

**Figure 2:**
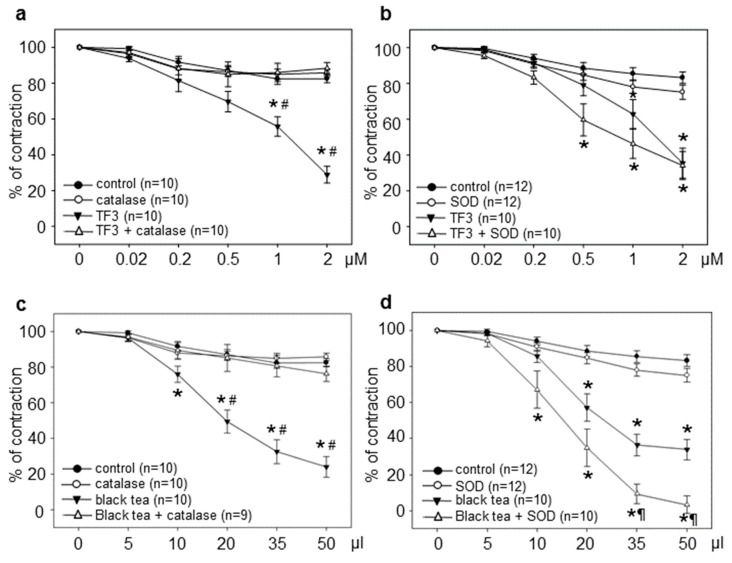
Catalase but not SOD prevented black tea polyphenol-induced vasorelaxation. Vasorelaxation was stimulated with cumulative doses of theaflavin-3,3’-digallate (TF3) (**a,b**) or black tea (**c,d**) with or without 200 U/mL catalase or 500 U/mL superoxide dismutase (SOD). Graphs show relaxation expressed as the percentage of maximal phenylephrine-induced vasoconstriction. Control rings received the same amount of water (control) or antioxidant enzymes alone. Data are means ± SEM of the indicated number of experiments. * *p* < 0.05 compared to the control; # *p* < 0.05 compared to treatment + catalase; ¶ *p* < 0.05 compared to black tea.

To extend our understanding of tea-induced vasodilation ex vivo, we also included black tea polyphenols. TF3 represents a major constituent of black tea and was shown to stimulate NO-dependent vasodilation [19]. TF3-induced vasorelaxation was completely inhibited by catalase (Figure 2a). In contrast, pretreatment with SOD resulted in a moderate increase in TF3-induced vasodilation. This effect was also observed in the control rings (Figure 2b). Black tea stimulated a strong vasorelaxation in aortic rings, which was, however, completely prevented by catalase (Figure 2c). SOD significantly amplified black tea-induced vasodilation, and also resulted in a slight reduction in PE-induced precontraction in the control rings (Figure 2d). Treatment with 50 µL of black tea resulted in a final concentration of 0.06 μM TF3 in the aortic rings. 

### 3.2. Rapid Degradation of Tea Polyphenols

**Figure 3:**
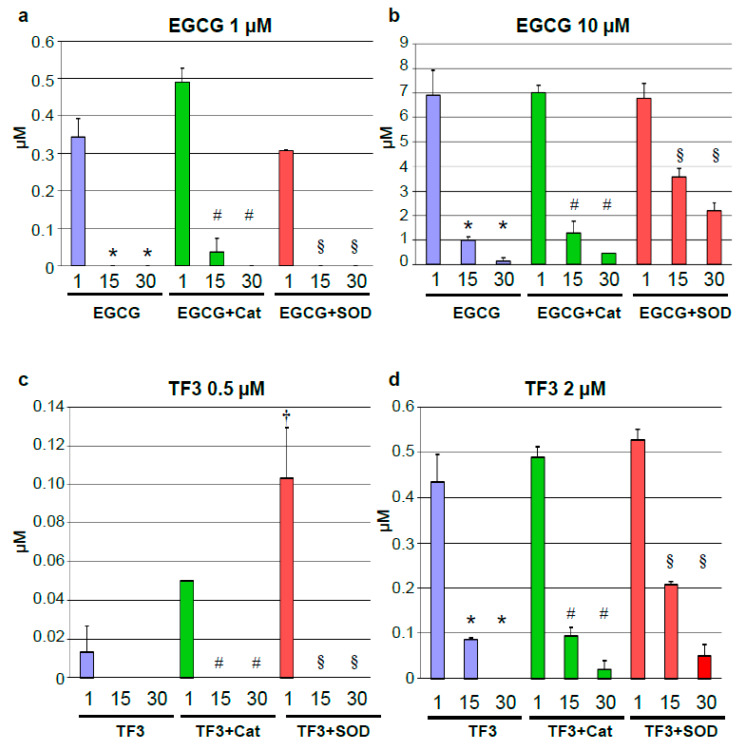
Decay of tea polyphenols. A single dose of EGCG (1 μM (**a**) or 10 μM (**b**) or of theaflavin-3,3’-digallate (TF3) (0.5 μM (**c**) or 2 μM (**d**) was applied. Aliquots of the Krebs–Henseleit solution were taken after the indicated time points and concentrations of EGCG and TF3 were determined by HPLC. Experiments were performed with or without 200 U/mL catalase or 500 U/mL superoxide dismutase (SOD). Data are means ± SEM from *n* = 3 experiments. * *p* < 0.05 compared to treatment without antioxidant enzymes after 1 min; # *p* < 0.05 compared to treatment + catalase after 1 min; § *p* < 0.05 compared to treatment + SOD after 1 min; † *p* < 0.05 compared to TF3 after 1 min.

Decomposition of tea polyphenols was reported under cell culture conditions [23]. We therefore measured concentrations of individual tea polyphenols at different time points after treatments with single doses. The chosen time points (1, 15 and 30 min) were deduced from the treatment intervals of the vasorelaxation experiments. EGCG at 1 and 10 µM were subject to rapid compound decay. A decline from the initial concentrations was observed already after 1 min. After 15 and 30 min, EGCG levels in the Krebs–Henseleit solution were below detection limits for 1 µM EGCG and almost zero for 10 µM EGCG (Figure 3a,b). Pretreatment with catalase had no major impact on EGCG decay. However, a stabilization by SOD was observed for the higher EGCG dose after 15 and 30 min (Figure 3b). TF3 at concentrations of 0.5 and 2 µM was also rapidly degraded. It was not detectable anymore after 15 (0.5 µM) and after 30 min (2 µM). Catalase and SOD had a minor impact on TF3 decay, though a trend towards compound stabilization was observed for SOD (Figure 3c,d).

### 3.3. Decay of Tea Polyphenols Is Associated With Generation of Hydrogen Peroxide

**Figure 4:**
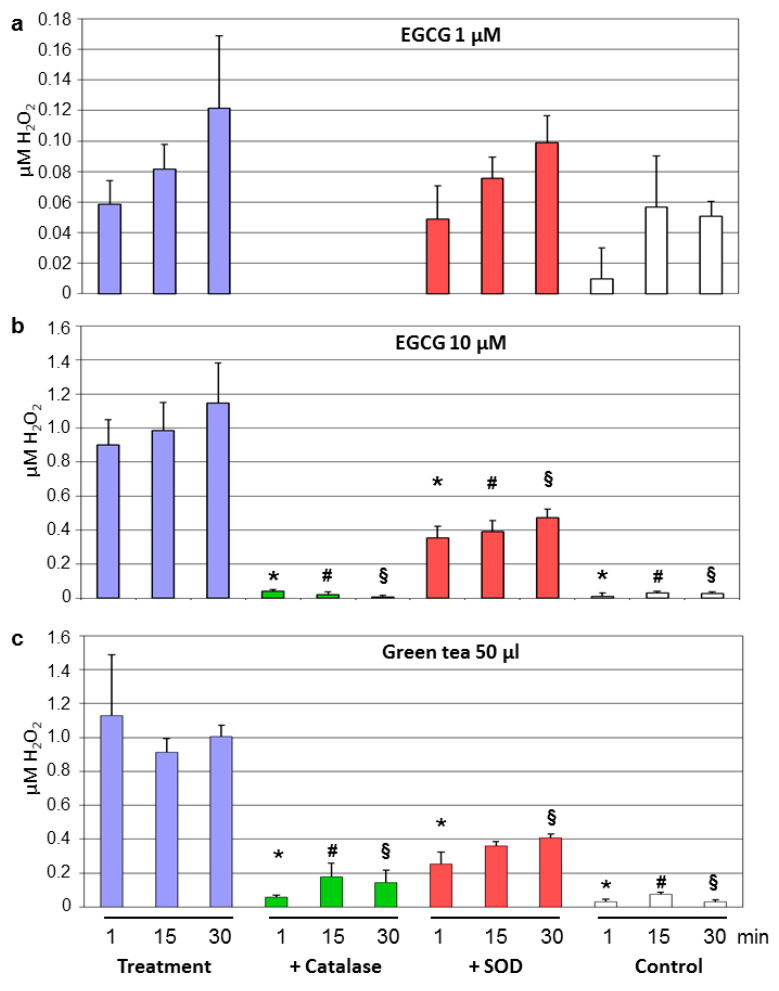
H_2_O_2_ production by EGCG and green tea. A single dose of EGCG (1 μM (**a**) or 10 μM (**b**) or of 50 μL green tea (**c**) was applied. Aliquots were taken after the indicated time points and concentrations of hydrogen peroxide in the Krebs–Henseleit buffer were determined. Experiments were performed with or without 200U/mL catalase or 500U/mL superoxide dismutase (SOD). Krebs–Henseleit buffer without any treatment served as controls. Data are means ± SEM from *n* = 4 experiments. * *p* < 0.05 compared to treatment without antioxidant enzymes after 1 min; # *p* < 0.05 compared to treatment without antioxidant enzymes after 15 min; § *p* < 0.05 compared to treatment without antioxidant enzymes after 30 min. The H_2_O_2_ levels for 1 μM EGCG + catalase were below the detection limits.

Tea polyphenol-induced production of H_2_O_2_ in cell culture media was described [27]. We therefore measured levels of hydrogen peroxide in the Krebs–Henseleit solution after different time points of a single dose of individual tea polyphenols or of green and black tea. Samples without treatments served as the controls. 1 µM EGCG produced low amounts of H_2_O_2_ over time. Generation of H_2_O_2_ was completely prevented by catalase and slightly decreased by SOD (Figure 4a). These effects were much more pronounced at 10 µM EGCG, a concentration which produced strong vasorelaxation in the aortic rings. Around 1 µM of hydrogen peroxide was generated by 10 µM EGCG over time (Figure 4b). Similar concentrations of H_2_O_2_ were produced by 50 µL of green tea (Figure 4c). H_2_O_2_ production by green tea and EGCG was strongly reduced by catalase and to a lesser extent by SOD (Figure 4b,c).

**Figure 5:**
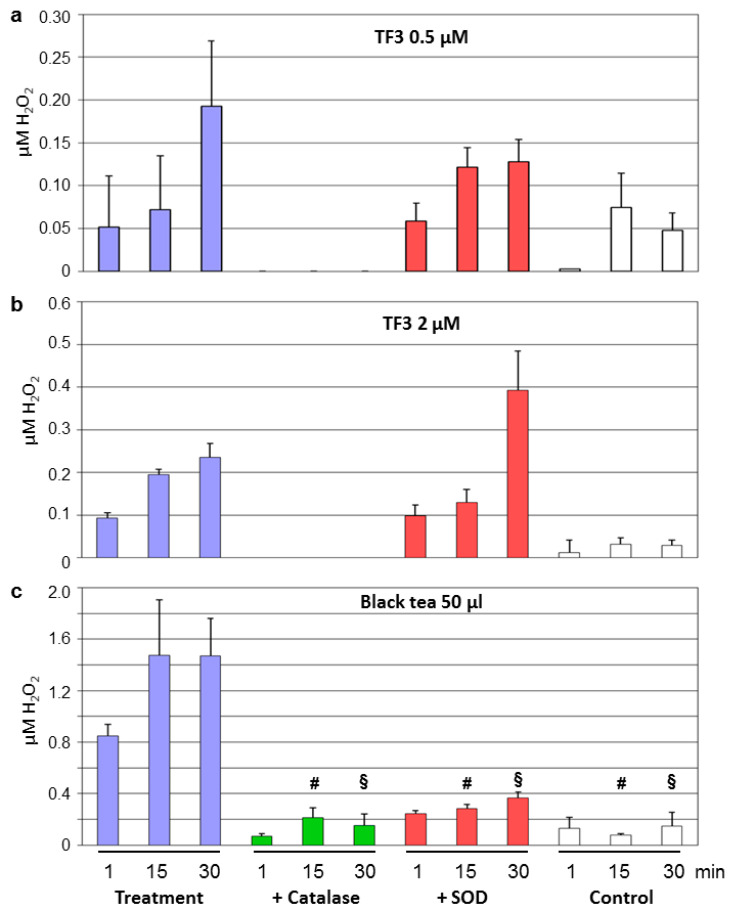
Black tea polyphenols generate H_2_O_2_. A single dose of TF3 (0.5 μM (**a**) or 2 μM (**b**) or of 50 μL black tea (**c**) was applied. Aliquots were taken after the indicated time points and concentrations of hydrogen peroxide in the Krebs–Henseleit buffer were determined. Experiments were performed with or without 200 U/mL catalase or 500 U/mL superoxide dismutase (SOD). Krebs–Henseleit buffer without any treatment served as controls. Data are means ± SEM from *n* = 3 experiments for TF3 and *n* = 4 for black tea. # *p* < 0.05 compared to treatment without antioxidant enzymes after 15 min; § *p* < 0.05 compared to treatment without antioxidant enzymes after 30 min. The H_2_O_2_ levels for 0.5 and 2 μM TF3 + catalase were below the detection limits.

Both concentrations of TF3 (0.5 and 2 µM) increased H_2_O_2_ levels above the control. Catalase completely inhibited H_2_O_2_ production, while SOD was without a consistent effect (Figure 5a,b). 2 µM TF3 produced less H_2_O_2_ compared to 10 µM EGCG (Figure 4b and Figure 5b). Treatment with black tea (50 µL) resulted in a substantial increase in H_2_O_2_ levels that was strongly suppressed by both catalase and SOD (Figure 5c). The amount of generated H_2_O_2_ was comparable between green and black tea (Figure 4c and Figure 5c).

### 3.4. Hydrogen Peroxide in Low Concentrations Stimulates NO-Dependent Vasorelaxation

**Figure 6:**
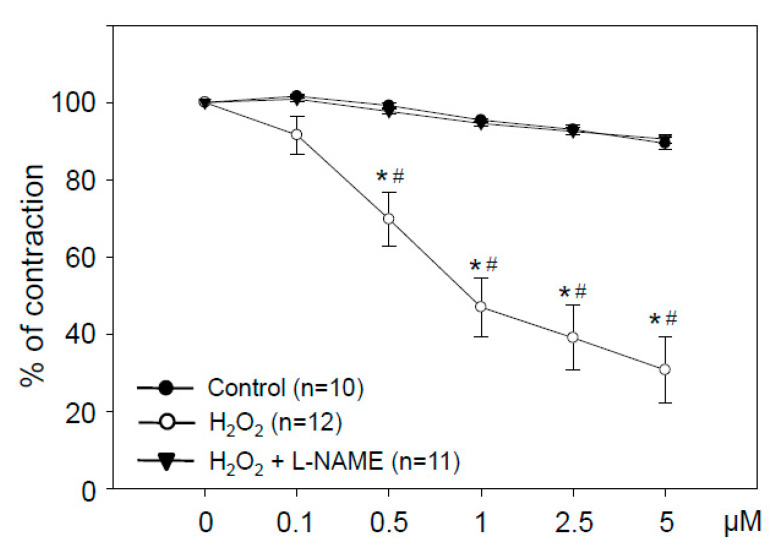
Hydrogen peroxide at low concentrations induces NO-dependent vasodilation. Aortic rings were treated with the indicated cumulative doses of H_2_O_2_. Selected rings were pretreated with the NOS-inhibitor N-nitro-L-arginine methyl ester (L-NAME, 100 μM) before contraction by phenylephrine. Graphs show relaxation expressed as percentage of maximal phenylephrine-induced vasoconstriction. Control rings received the same amount of water. Data are means ± SEM of the indicated number of experiments. * *p* < 0.05 compared to the control; # *p* < 0.05 compared to H_2_O_2_ + L-NAME.

H_2_O_2_ at 0.1 to 5 µM stimulated a pronounced concentration-dependent vasorelaxation. This vasodilation was completely prevented by the nitric oxide synthase inhibitor L-NAME (Figure 6). H_2_O_2_-induced vasodilation reached statistical significance at 0.5 µM.

## 4. Discussion

The major finding of our study is that the vasodilatory properties of EGCG and TF3, as well as of the whole beverages green and black tea, are mediated ex vivo by the production of hydrogen peroxide. The levels of hydrogen peroxide generated during the decay of the tea polyphenols result in NO-dependent vasodilation.

Tea polyphenol-induced vasodilation in isolated vessels was described previously [28,29,30]. Both green and black tea and isolated tea polyphenols activate endothelial NO synthase (eNOS) and NO production in endothelial cells [20,31,32]. In addition, EGCG failed to induce vasorelaxation in aortic rings from eNOS knockout mice [33]. These observations point to a mechanism of endothelial- and NO-dependent vasodilation by tea polyphenols. On the other hand, tea polyphenols were shown to produce ROS in cell culture media [34]. There is an ongoing debate about pro- versus antioxidant activities of tea polyphenols [35]. Pro- as well as antioxidant properties of tea polyphenols have been shown in vitro [34,36]. Tea extracts and catechins can be oxidized by molecular oxygen [37]. As a consequence, the involvement of ROS in EGCG-induced eNOS activation and vasodilation have been described [38,39]. On the other hand, tea polyphenols were shown to generate low amounts of superoxide [40]. In our study, we noticed a slight increase in tea polyphenol-induced vasodilation in the presence of SOD. Superoxide reacts with NO to peroxynitrite and thereby reduces NO-dependent vasodilation [41], explaining the amplification of tea-induced vasodilation by SOD.

In contrast, tea polyphenol-induced vasodilation was completely prevented in the presence of catalase. Inhibition of vasorelaxation by catalase suggests the involvement of hydrogen peroxide. Indeed, we detected increasing levels of H_2_O_2_ in the Krebs–Henseleit solution with the duration of the experiment. Whereas hydrogen peroxide concentrations increased over time, levels of polyphenols diminished. Many polyphenols, including green tea catechins, were shown to produce hydrogen peroxide under cell culture conditions and in phosphate-buffered saline (PBS) [27,40]. Hydrogen peroxide is able to affect numerous cell signaling pathways. Phosphorylation of eNOS and protein kinase B (Akt) by EGCG in endothelial cells was mediated via generation of H_2_O_2_ [38]. In addition, vasodilation in rat coronary arterioles was stimulated by hydrogen peroxide. This vasorelaxation involved oxidation of intracellular thiol groups and phosphorylation of the redox-sensitive p38 MAP kinase [42]. In rat mesenteric arteries, the vasodilatory effects of hydrogen peroxide were attributed to stimulation of voltage-gated K^+^ channels [43]. However, the above studies used high concentrations of hydrogen peroxide (10 µM to 10 mM). In our study, levels as low as 500 nM H_2_O_2_ resulted in NO-dependent vasorelaxation in rat aortic rings. This corresponded with the H_2_O_2_ levels generated during tea polyphenol degradation.

Which mechanisms could contribute to tea polyphenol-induced production of hydrogen peroxide in our experimental model? We observed a rapid decay in tea polyphenols, which was only partially prevented by catalase and SOD. The stability of polyphenols is highly pH-dependent [22]. EGCG was degraded in a Krebs–Ringer bicarbonate buffer (similar in composition to our Krebs-Henseleit solution at pH 7.4) within a few minutes [23]. The black tea polyphenol theaflavin was also found unstable under conditions of higher pH [25], underscoring our findings on the similar instability of green and black tea polyphenols. In addition to higher pH values, the Krebs–Henseleit buffer is constantly gassed with carbogen (mixture of 5% CO_2_ and 95% O_2_), to maintain physiological functionality of the explanted organs. These experimental conditions result in high oxygen pressure throughout the experiments. The major reason for instability of tea polyphenols thus appears to be oxidative modification. It was reported that EGCG undergoes auto-oxidation and subsequent epimerization [44], which is associated with production of hydrogen peroxide.

Instabilities of polyphenols in vitro raise the question about biological effects in vivo. Blood contains high levels of antioxidant defense enzymes that can prevent oxidation and/or neutralize ROS generated during potential compound decay. Whereas auto-oxidation of EGCG with formation of oxidation products and various dimers occurred in Tris-buffer, no auto-oxidation products were detected in the plasma of mice after treatment with EGCG for 3 days [45]. Human studies revealed discrepancies between the in vitro and in vivo vasorelaxant effects of tea polyphenols. Recently, we observed an improvement of FMD after consumption of green tea (containing 200 mg of EGCG) in humans. However, the same amount of isolated EGCG had no effect, despite high EGCG plasma levels after intervention [21]. This indicates that EGCG does apparently not contribute to tea-induced improvement of endothelial function in vivo. The vasorelaxant effects of tea polyphenols in isolated organs and of NO production in cultured endothelial cells are most likely induced by the experimental conditions.

## 5. Conclusions

Our study shows that tea polyphenol-induced vasodilation in organ bath models is characterized by a compound decay with production of hydrogen peroxide over time. Hydrogen peroxide in turn stimulates NO-dependent vasodilation. The results point to the limitations of the ex vivo model, involving higher pH values and carbogen to maintain the physiological functionality of the explanted organs. These findings may not apply in vivo, where antioxidant defense enzymes may confer a higher stability to tea polyphenols.
